# Assessing the human footprint on the sea-floor of coastal systems: the case of the Venice Lagoon, Italy

**DOI:** 10.1038/s41598-019-43027-7

**Published:** 2019-04-29

**Authors:** Fantina Madricardo, Federica Foglini, Elisabetta Campiani, Valentina Grande, Elena Catenacci, Antonio Petrizzo, Aleksandra Kruss, Carlotta Toso, Fabio Trincardi

**Affiliations:** 1 CNR-National Research Council, ISMAR- Institute of Marine Sciences in Venice, Castello 2737/f, 30122 Venice, Italy; 2 CNR-National Research Council, ISMAR-Institute of Marine Sciences in Bologna, Via Gobetti, 101, 40129 Bologna, Italy

**Keywords:** Environmental impact, Geomorphology

## Abstract

Coastal systems are among the most studied, most vulnerable, and economically most important ecosystems on Earth; nevertheless, little attention has been paid, so far, to the consequences of human activities on the shallow sea-floor of these environments. Here, we present a quantitative assessment of the effects of human actions on the floor of the tidal channels from the Venice Lagoon using 2500 kilometres of full coverage multibeam bathymetric mapping. Such extended dataset provides unprecedented evidence of pervasive human impacts, which extend far beyond the well known shrinking of salt marshes and artificial modifications of inlet geometries. Direct and indirect human imprints include dredging marks and fast-growing scours around anthropogenic structures built to protect the historical city of Venice from flooding. In addition, we document multiple effects of ship traffic (propeller-wash erosion, keel ploughing) and diffuse littering on the sea-floor. Particularly relevant, in view of the ongoing interventions on the lagoon morphology, is the evidence of the rapid morphological changes affecting the sea-floor and threatening the stability of anthropogenic structures.

## Introduction

Human activity has profoundly altered the global environment to such an extent that a worldwide debate is ongoing within the scientific community to determine whether we are now in a new geological Epoch, called the Anthropocene^1,2^, or not^3,4^. The term Anthropocene denotes a new human-dominated geological time unit (still waiting to be acknowledged officially) in which humans are recognized as a geological factor, for their activities are radically changing the Earth System state and functioning^5^. The magnitude and increase rate of such human-induced changes has become particularly dramatic since 1950, an abrupt shift often indicated as the ‘Great Acceleration’^6,7^. This acceleration was observed by studying the trends from the 1750 to 2010 of the main socio-economic indicators, such as population, economic growth, use of non renewable resources, urbanization, globalization, transport and communication, and the indicators for the structure and functioning of the Earth System.

Indeed, the human-induced changes in the Anthropocene profoundly concern the oceans through direct or indirect actions affecting a large fraction (more than 40%) of the marine ecosystems^8^. Beyond the warming of the ocean’s upper layers, sea-level rise, acidification and changes in ocean circulation^9^, the biophysical interaction with sea-floor environments (covering 70% of the Earth), contributes significantly to global ecosystem functions and services^10^. The impact of human activities on the sea-floor, such as trawling, dredging and dumping, have been documented in coastal and shelf environments and in deep sea areas: extensive bottom trawling following the industrialization of fishing fleets, in particular, has been shown to be an important driver in the sediment dynamics and the global deep seascape evolution^11–15^.

In shallow areas substantial transformation of coastal landscapes has occurred globally in response to urbanization sprawling toward the intertidal zone and in near shore estuarine environments and to anthropogenic hydraulic engineering, especially huge dams^11,16–20^. The proliferation of a variety of built structures, such as breakwaters, seawalls, jetties and pilings is increasingly impacting on the coastal ecosystems^21–23^. This “coastal urbanization” caused by artificial structures being developed in coastal environments has widespread and yet poorly known ecological consequences^24^.

**Figure 1:**
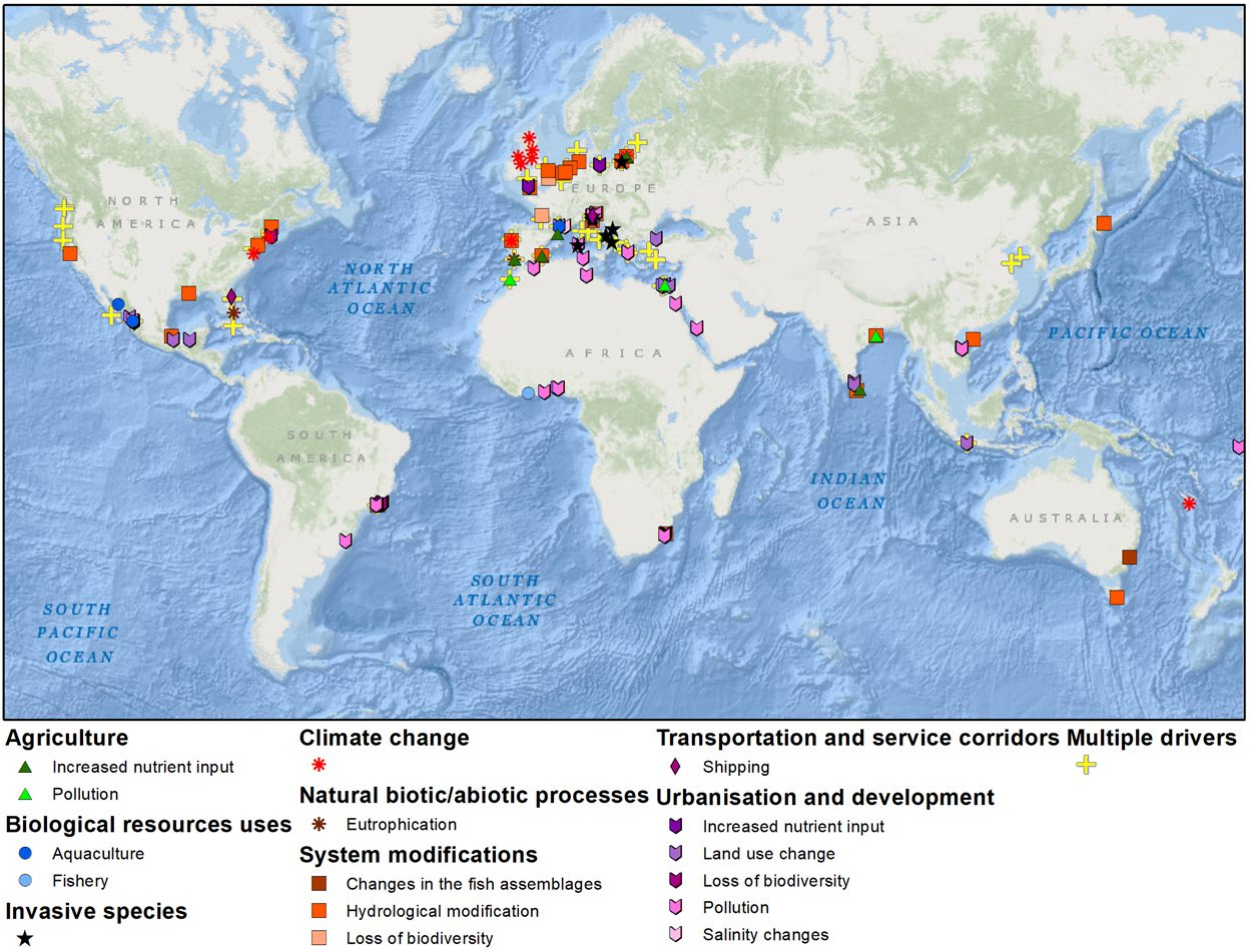
Summary of multiple human drivers altering coastal lagoons over the world (see Supplementary Material).

Coastal wetlands are valuable ecosystems^25^ extremely impacted by humans^26^. Beside urbanization, these areas are also threatened by sea-level rise^27,28^, land subsidence^11,29^ and the increase of flood and storm events^30,31^. Globally, in coastal wetlands a rapid decline of salt marsh areas has been documented (25% in the last two centuries), mostly driven by land reclamation and other human activities^32–34^. The impacts on coastal wetlands induced by a variety of human activities have been studied all over the world as it is shown in Fig. 1. However, none of the 152 reviewed studies published between 1991 and 2018 focused on the assessment of the human footprint on the sea-floor of coastal wetlands.

The recent technological development of bathymetric instruments allows a new approach to the knowledge of the hidden bottom of very shallow water environments. This approach includes for the first time the possibility of quantifying human impacts, beyond the information currently achieved in assessing environmental quality by coring through bottom sediments. Multibeam echo-sounders (MBES) can collect simultaneously geo-referenced bathymetric and backscatter data and guarantee high-resolution and full seabed coverage, opening new frontiers in the field of mapping sea-floor morphologies, substrates, mobile bedform fields and habitats^35,36^.

The aim of this study is to show how the MBES mapping can be used to assess quantitatively the extent and the impacts of human activities on the sea-floor and to identify marine litter hot-spots in coastal areas. A dual-head MBES dataset of the entire network of tidal channels and inlets of the lagoon surrounding the historical city of Venice (Italy) provided, with up to 5 cm resolution, a completely novel three-dimensional view of the channel sea-floor^37^, disclosing a hidden world of human modifications and diffused littering of the Venice Lagoon sea-floor, revealed with unprecedented detail. The high resolution and the extensive dataset allowed the evaluation of all submerged human pressures and the estimate of their long-lasting consequences on sea-floor morphology and, consequently, on its habitat properties.

## The Venice Lagoon and the human activities over time

**Figure 2:**
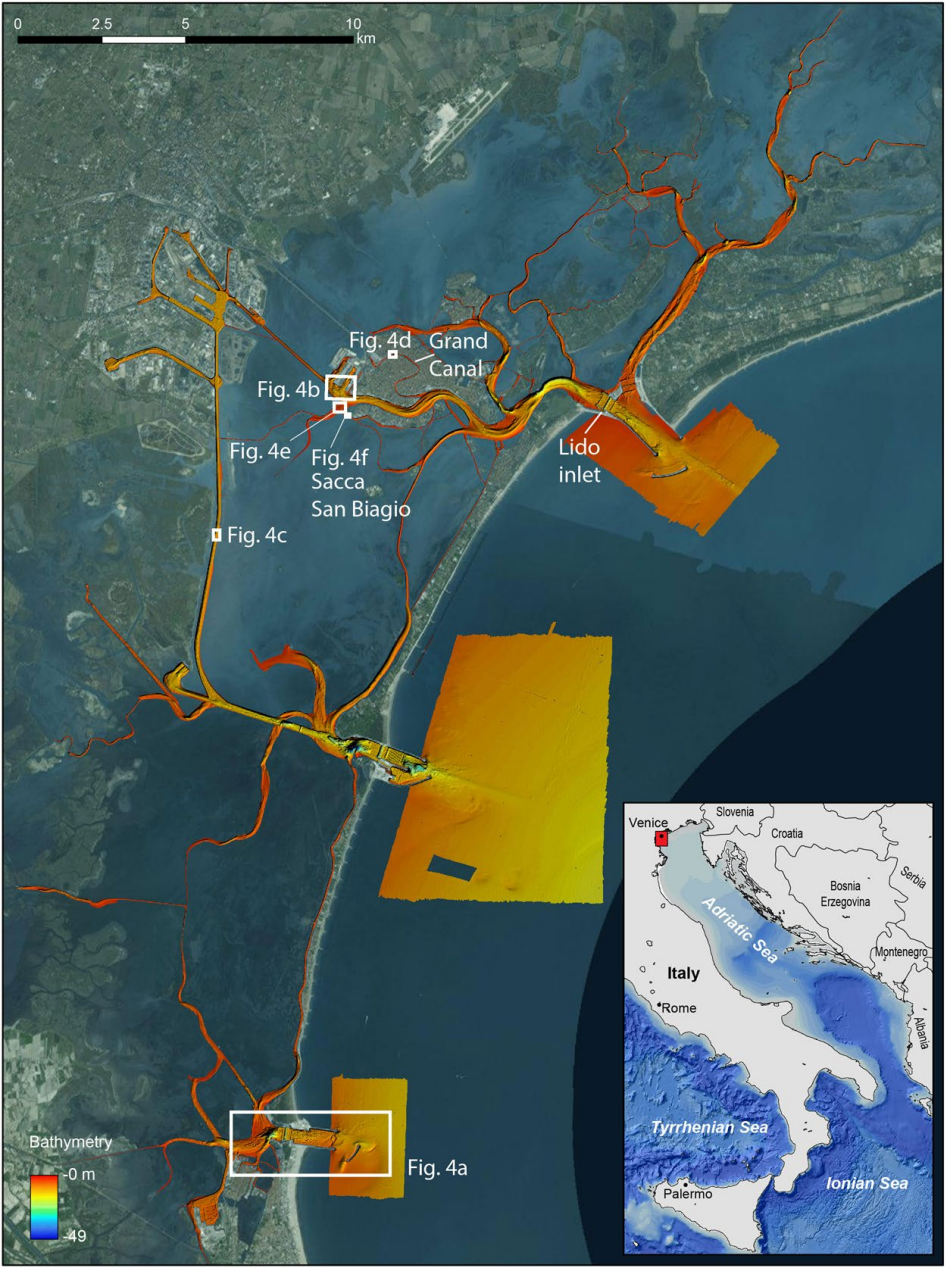
The Venice Lagoon and the bathymetry of the tidal channels. The white polygons indicate the locations of the anthropogenic features described in the text. Satellite image source: Esri DigitalGlobe, GeoEye, i-cubed, USDA, USGS, AEX, Getmapping, Aerogrid, IGN, IGP, swisstopo, and the GIS User Community, https://services.arcgisonline.com/ArcGIS/rest/services/World_Imagery/MapServer.

The Venice Lagoon (Italy) is the largest coastal transitional ecosystem in the Mediterranean and, at the same time, one of the UNESCO World Cultural and Natural Heritage sites. It is characterized by a maze of channels (maximum depth exceeding 15 m), which cut across a large area of shallow waters (average depth of 1 m), fens and salt marshes. Today the total surface of the lagoon is 550 km^2^: 390 km^2^ of open lagoon including 40 km^2^ of tidal channels, 70 km^2^ of salt marshes, and 90 km^2^ of fish farms. Three inlets (from north to south: Lido, Malamocco and Chioggia), connect the lagoon to the Adriatic Sea (Fig. 2).

The morphology and extent of the Venice Lagoon has been strongly influenced by humans since remote times: (i) the islands within the northern Lagoon have been inhabited since Roman times and up to the Medieval Age^38^; (ii) the city of Venice was one of the largest in Europe with a population of 100,000 inhabitants by the end of the 13th century^39^; (iii) the city is now receiving more than 25 million visitors per year. The Venice Lagoon represents a paradigmatic case of ecosystem alteration in the Anthropocene, since human activities continuously modified the environment through the centuries: the diversion of its major tributaries outside the lagoon to prevent sedimentation in marginal areas (from the XV to the XVII century); the construction of rigid defenses to protect the barrier islands from storm waves (1740–1782); the construction of successive sets of jetties at the inlets (1808–1927); the land reclamation for urban and industrial development (1927–1960); subsidence induced by ground water and natural gas extractions (about 9 cm from 1930 to 1970); the stabilization or the construction of artificial salt marshes (since the 1990s); and, lately, the construction of mobile barriers (MOSE Project) at the inlets for flood protection of Venice (since 2003). All such human imprints are visible or can be deduced by comparing modern and historical maps^40–42^ (see supplementary material). Similar changes on the sea-floor of the lagoon are not as easy to document except for the dredging of a deep canal for oil tankers (“Canale dei petroli”; 1960–1970) and the substantial deepening and land loss (50% salt marsh surface loss) documented by comparing sea-floor cartographies of successive ages^43,44^. The quantitative comparison of bathymetric surveys taken in 1927, 1970 and 2002 indicates that most of the erosion affected the central and southern portions of the lagoon. Between 1927 and 1970, the salt marsh surface shrunk, with an acceleration of the erosive processes from 1970 to 2002, when a general expansion of subtidal flats (areas deeper than 1 m) occurred mostly in the central basin. An estimated net sediment loss of 110 Mm^3^ between 1927 and 2002 corresponds to an average annual loss rate of 0.5 Mm^3^. From 1970 and 2002, this rate of sediment export increased to 0.8 Mm^3^ ^43,44^.

The three inlets have been significantly modified for the construction of a complex array of large mobile barriers (MOSE system) to protect the city from floods (“high water events”). In fact, the frequency and impact of flooding events in Venice is likely to increase given relative sea level rise and climate change. Once in full operational mode, the MOSE system might substantially affect the lagoon hydrodynamics and, consequently, sediment transport and sediment balance. More generally, the MOSE system is a coastal protection device against storm surges which is potentially efficient only in a frame of overall mean sea level rise of 25 cm by the end of the Century, a scenario that is questioned by the most recent scientific literature. A discussion on the implicit assumptions and limitations of the MOSE solution for the high water in Venice can be found in Trincardi *et al*.^45^.

## Human footprint on the sea-floor

**Figure 3:**
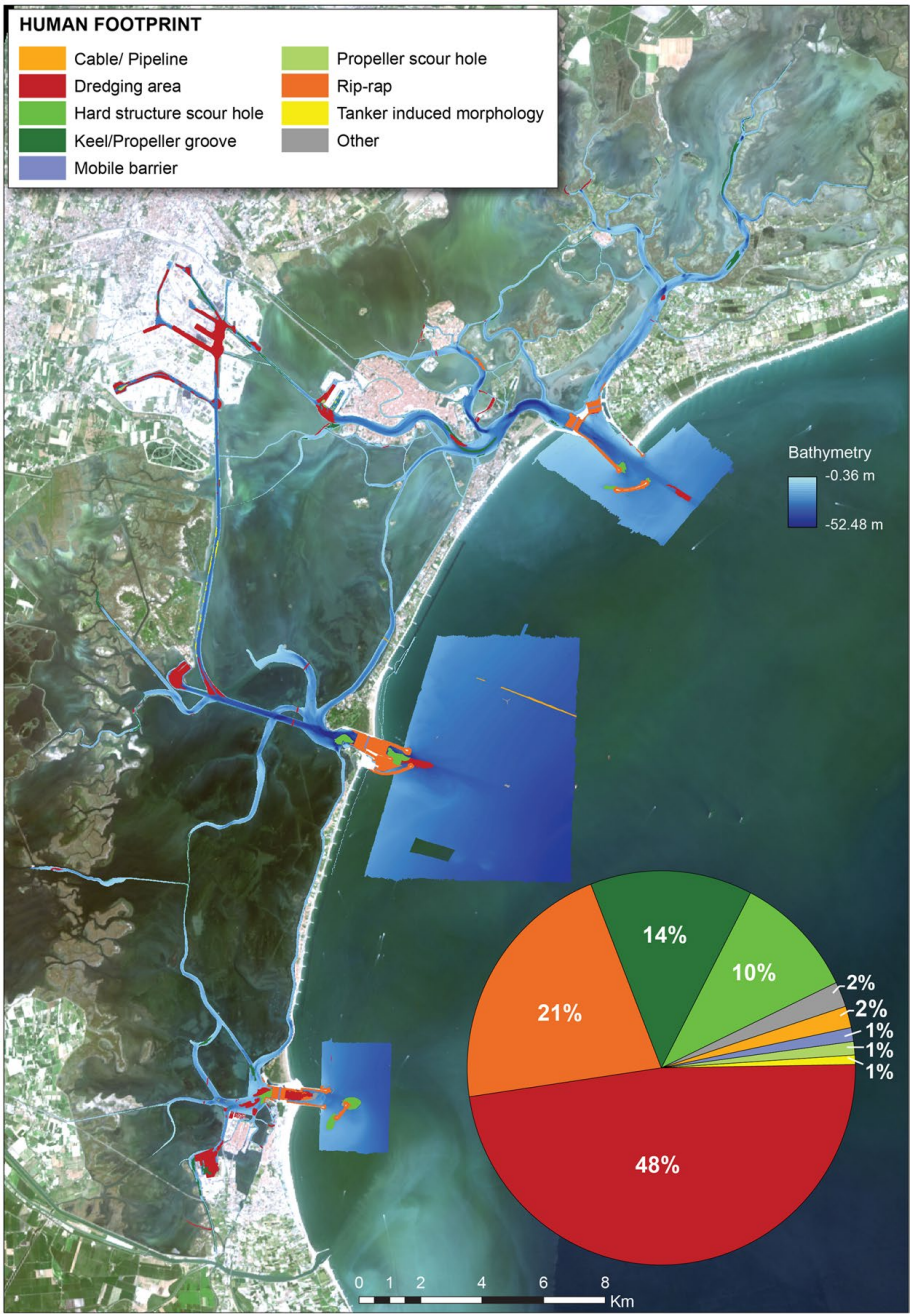
Human footprint on the sea-floor and pie chart illustrating the relative extent of the distinctive anthropogenic morphological features. Satellite image source: Esri DigitalGlobe, GeoEye, i-cubed, USDA,USGS,AEX,Getmapping, Aerogrid, IGN, IGP, swisstopo, and the GIS User Community, https://services.arcgisonline.com/ArcGIS/rest/services/World_Imagery/MapServer.

The signs of the human intervention detected in the Venice Lagoon have been classified as direct, where human action actively modified the morphology of the lagoon sea-floor, and indirect, where new bottom morphologies emerge as the reaction of the tidal system to a human perturbation. By analyzing the high resolution digital elevation model (DEM) obtained from the MBES data (Fig. 3), we assess the extent of the human footprint on the sea-floor (where the human footprint is here defined as in Kenny *et al*.^46^).

The sea-floor modified by human activities is equal to 7.8 km^2^ (ca. 24%) of the total tidal channel surface (Fig. 3). The sea-floor is morphologically modified mostly by dredging (3.72 km^2^), by the presence of the rip-rap used for the structures at the inlets (1.67 km^2^), by the keel/propeller grooves (1.06 km^2^), by the hard structure and related scour holes (0.8 km^2^), by a maze of cables and pipelines (0.14 km^2^), by the lodgements of the MOSE mobile barriers (0.09 km^2^), by the propeller scour holes (0.09 km^2^), and by possible ship induced gullies dissecting the slopes of the Canale dei Petroli (0.08 km^2^).

### Dredging areas

**Figure 4:**
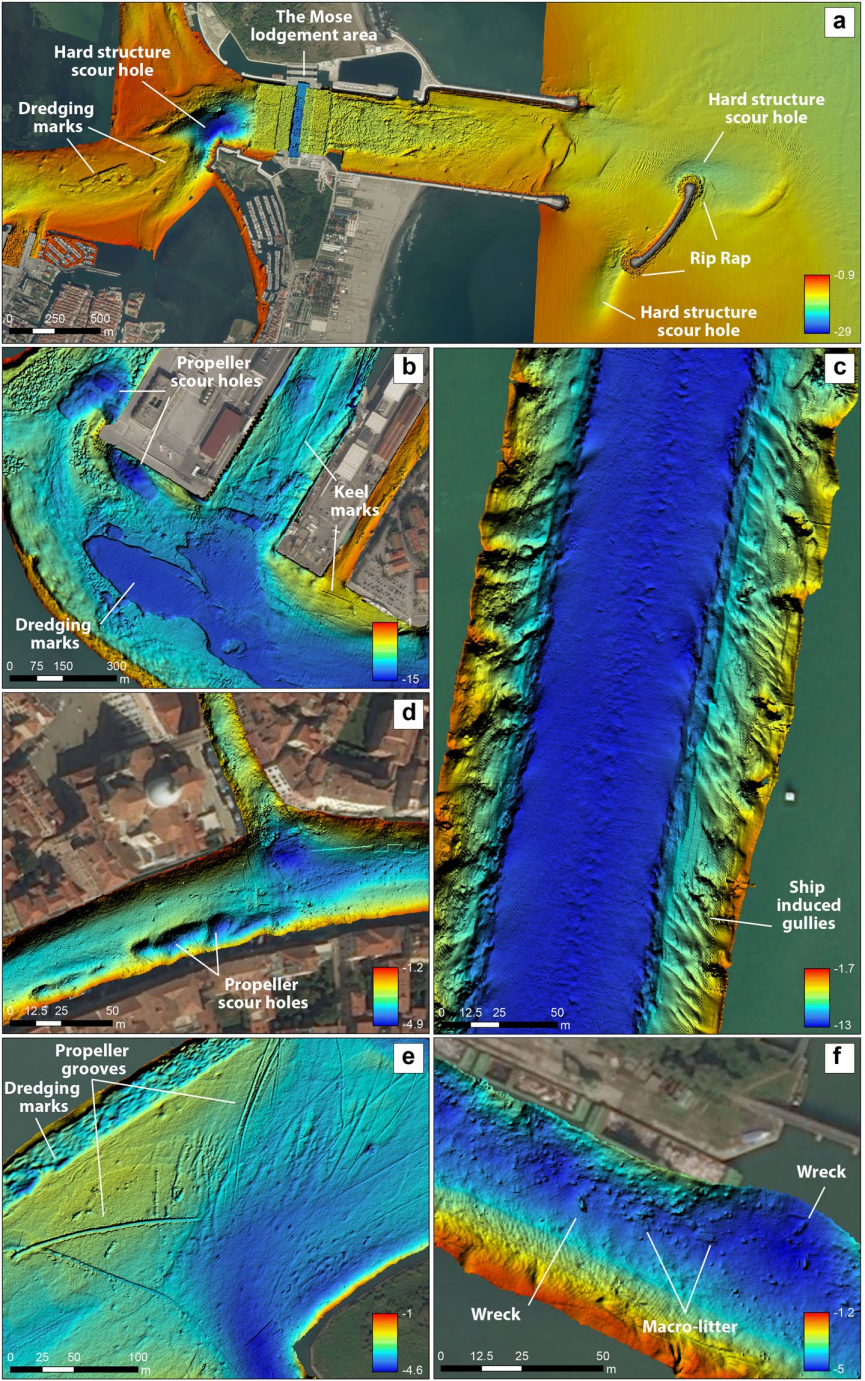
High resolution bathymetry maps (0.2 m) of (**a**) the southernmost inlet (Chioggia) of the Venice Lagoon with dredging, rip-rap, the MOSE lodgment areas and scours rapidly formed at the breakwater tips; (**b**) The Tronchetto cruise-ship harbour with dredging marks, propeller scour holes and keel grooves; (**c**) zoom of the Malamocco-Marghera industrial canal, where dredging is carried out in the central part and gullies dissect the channel sides; (**d**) scour holes typically 1.5 m deep induced by water busses at one docking station on the Grand Canal; (**e**) dredging area on the side of a shallow canal and evidence of propeller grooves on the lagoon floor; (**f**) garbage distributed on the floor of a Venice Lagoon channel showing wrecks, containers and smaller debris, including thrown-away rubber fenders. Satellite image source: Esri DigitalGlobe, GeoEye, i-cubed, USDA,USGS,AEX,Getmapping, Aerogrid, IGN, IGP, swisstopo, and the GIS User Community, https://services.arcgisonline.com/ArcGIS/rest/services/World_Imagery/MapServer.

Dredging areas were identified in the inlets, in the main navigation channels and in the Tronchetto harbour (Figs 3 and 4a–c). It is possible that dredging areas be underestimated because tidal currents may have healed out the sea-floor morphology in the oldest cases. Nowadays, estuarine ports are a crucial factor for the blue growth economy^8^. The global trade network is continuously growing together with the number of navigating commercial ships steadily increasing and requiring more intensive dredging of the waterways to the harbours, often sheltered in the internal part of lagoons or estuaries. As a consequence, over the past decade the global dredging market increased from $ 5.3 bn in 2000 to $ 14.7 bn in 2011, according to the International Association of Dredging Companies^47^. The environmental impact of dredging and dredge-spoil dumping on the sea-floor integrity has been recognized since the 1960s and mainly ascribed to the physical removal of substratum and benthic communities from the seabed, the resuspension and deposition of the dumped material and consequent increase of the level of turbidity, organic and metal compounds in the water and dredged sediment^48–52^. Moreover, in tidal environments the channel deepening by dredging can impact physical processes such as the strength of the estuarine exchange flow and related sediment transport, the horizontal salinity gradient, and the general tidal dynamics^53^.

Dredging has played a major role in the variation of the lagoon bathymetry particularly in the deepening of its central part^44,54^. Establishing a rate of change for the dredging activity, though, cannot be based only on morphological evidence, because successive dredging events can be superimposed in a given area. A recurrence of dredging events in a critical area, such as a navigation canal that silts up in response to the erosion of the adjacent shoals should rely only on data from the harbour authority that are typically restricted.

### Rip-rap

**Figure 5:**
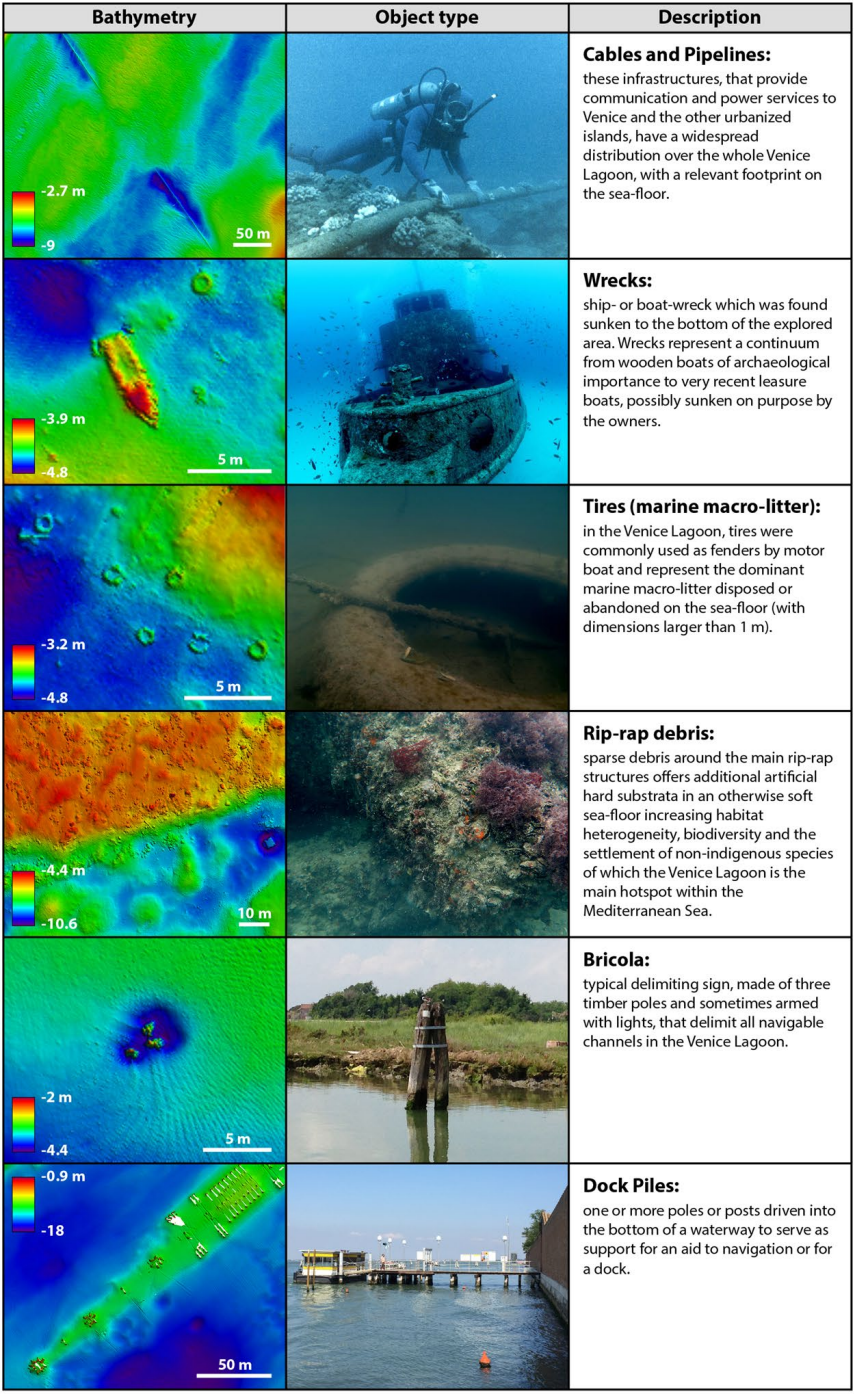
High resolution bathymetry (0.05 m) (obtained from the CNR-ISMAR data using the software CARIS HIPS and SIPS v.9, http://www.teledynecaris.com/en/products/hips-and-sips/) and images relative to the main categories of relatively small dimension anthropogenic morphologies found on the sea-floor. The copyright of the picture of the cable belongs to the CTBTO Preparatory Commission; this picture has the license CC BY 2.0 (https://creativecommons.org/licenses/by/2.0/) and was downloaded without modifications from the link https://www.flickr.com/photos/ctbto/3816716741; the picture of the wreck was downloaded from the link https://commons.wikimedia.org/wiki/File:MV_Rozi_01.jpg; the pictures of the submerged tyre and rip-rap are by Riccardo Fiorin, Laguna Project Snc, whereas the pictures of the briccola and the dock piles were taken by the main author.

Rip-rap revetments are commonly adopted worldwide to armour shorelines and to build jetties, seawalls or bulkheads, to ensure navigation and to protect the shoreline against erosion. Rip-rap is present on the sea-floor in the Venice Lagoon in correspondence to the long jetties built in the 19th and at the beginning of the 20th century to stabilize the inlets^55^. Our data highlight the presence of rip-rap at the inlets and in the channels immediately adjacent to the inlets (Figs 3 and 4a), as well as rip-rap debris (Figs 4a and 5). The mapped areas include the rip-rap utilized since 2002 within the MOSE project to: (a) build breakwaters at the seaward side of the inlets; (b) build the artificial island within the Lido Inlet; (c) armour the sea-floor in proximity of the mobile barriers lodgements in the three inlets to prevent their filling by sediments (Figs 3 and 4a). Rip-rap represent artificial hard substrata in an otherwise soft and mobile sea-floor and therefore increase habitat heterogeneity potentially enhancing biodiversity^21^, including the capability to host non indigenous species^56^. This is particularly relevant for the Venice Lagoon that represents the main hotspot for non-indigenous species within the Mediterranean Sea^57^. The number of hard coastal-defense structures is likely to increase worldwide in response to global mean sea-level rise and a possibly augmented intensity and frequency of large storms^22,58^. These artificial structures can consequently become the dominant intertidal and shallow subtidal habitat of coastal waters in proximity of urbanized centers^21,59–63^.

### Keel/propeller grooves

The keel/propeller grooves are anthropogenic erosional features, produced by ship keels or propellers getting stuck at or ploughing the channel bottom, where the draft is higher than the channel depth. These peculiar tool marks appear as linear grooves a few meters wide and up to hundreds of meters long and tend to be deeper and more enhanced as the channel floor shoals (Fig. 4b and e). The smallest keel/propeller marks are more frequently encountered at channel junctions where boats coming from a deeper channel are directed to a shallower one; such features are also observed where small boats attempt a dangerous navigation outside the limits of the channels that are typically marked by the “bricole” (the delimiting signs, made of three timber poles, that delimit all navigable channels in the Venice Lagoon; see also Fig. 5).

The waves generated by the small boat traffic could be relevant for tidal flat and salt marsh erosion especially in the northern lagoon^64–68^. The small boats, though, do not have the Automatic Identification System (AIS) traffic tracking and currently it is hard to monitor their movements and to relate them to the lagoon bathymetric variation.

### Hard structure scour holes

Large hard structure scour holes formed at the edge of the outer breakwaters built between 2005 and 2011 in front of each inlet to protect the mobile barrier against the action of storm waves (Fig. 4a). Anthropogenic hard structures are known to exert a constriction of the flow field typically leading to focused erosion around them, thus generating scours^69^. Scours occurring around breakwaters have been observed extensively^70–72^, and the mechanism behind their formation have been studied mainly on the basis of tank experiments^69,73–75^. The shape of the scours detected in the study area is very similar to the scour related to the effect of non breaking waves described by Sumer and Fredsoe^73^ at the head of a vertical breakwater. The detailed MBES bathymetry allowed the identification of these erosive features particularly evident outside the Chioggia inlet (Fig. 4a) where we estimated that waves and ebb currents in the order of 1 m s^−1^, eroded about 430’000 m^3^ of sediment in just 8 years.

### Cable and Pipelines

Cable and Pipelines are distributed over the whole Venice Lagoon providing services to Venice and all the smaller urbanized islands (Fig. 5). Submarine cables and pipelines have been laid in many parts of the global oceans^76^ and more than 95% of international communications are today routed via submarine fibre-optic cables^77^. In waters deeper than 200 m, the spatial extent of cables and pipelines is almost negligible with respect to other more extensive human impacts such as, for example, the areas impacted by bottom trawling^77^. By mapping the physical presence of cables and pipelines on the floor of the Venice Lagoon, our study shows that the footprint of such features is relevant, even not considering those that are entirely buried beneath the sea-floor.

### The MOSE mobile barriers

The structure to host the MOSE mobile barriers, under construction since 2003, have introduced additional and extensive anthropogenic modifications at the inlets of the Venice Lagoon that modify the whole lagoon hydrodynamics and morphodynamics^78^: the narrowing of the inlet sections designed to provide space for auxiliary infrastructures, like navigation locks and refuge harbours, altogether increased the flow velocity^79^. On the other hand, such modifications augmented the flow resistance, thus reducing the water exchange between the lagoon and the sea and increasing the amplitude of the major tidal costituents inside the lagoon^80^.

Satellite data for the piers rimming the inlet near the MoSE structure show quantitatively that the load of these new structures induces a subsidence rate up to 40 mm/year in some sectors of the inlet^29^. We surmise that comparable rates can be expected to affect also the sea-floor of the inlet loaded by the gates and their concrete lodgement cases. The interaction of these hard structures with tidal currents, wind waves and longshore drift has been poorly considered so far and can be quantified using time lapses of MBES data.

### Propeller scour holes

Propeller scour holes on channel flanks are ascribed to the erosion caused by the boat propeller-induced flow of water near the sea-floor (Fig. 4b and d). These erosional patterns are quite complex in docking areas where boats of highly variable size (and engine power) are frequent. Amongst the many channels of the lagoon, the Grand Canal, the main navigation channel in the city center of Venice (Fig. 1), is impacted by the traffic of water taxis, water buses, and other private small motor boats. A major, and more recent, sign of human impact on the sea-floor of the Grand Canal, as well as on the sea-floor of other navigated channels in the urban areas, is the presence of large elongated scours (up to 40 m long, 15 m wide and 1.5 m deep) generated by water buses (‘vaporetto’; Fig. 4d), and here documented for the first time. Similar but deeper and larger scours (up to 120 m long, 80 m wide and 3.5 m deep) in proximity to the cruise ship harbour are likely generated by the propellers of larger ships during docking operations (Fig. 4b). The seabed erosion by ship propellers is a well known phenomenon and occurs mainly in the berthing and unberthing manoeuvres of the ship^81^. Interestingly, in the case of the Grand Canal the depth of the scours is the same (1.5 m) in areas of frequent docking by water buses of all sizes and in the case of bus stops used at much lower frequency by only one water bus line. We suggest that the maximum depth of erosion coincides with a major change in soil composition and geotechnical strength corresponding to the subcrop of the alluvial plain consolidated sediment (Caranto in venetian dialect) on which lay the foundation of the entire city of Venice^82^.

### Ship induced gullies

The Malamocco-Marghera Industrial Canal, dug in 1970, presents steep and morphologically more complex flanks than any other natural channel in the Lagoon; we documented the presence of bedforms and possible gullies, a feature that is not shared by the typical sinuous channels of the lagoon. These gullies are perpendicular to the canal’s edges and oriented in the direction of the net transport of water induced by the passage ships in the canal (see Fig. 9 of Rapaglia *et al*.^54^). More than 3000 commercial vessels navigate through this industrial canal, leading to an estimated resuspension of 1.2 × 10^6^ metric tons of sediment per year^54^, contributing to significant erosion of shoals in the central lagoon in the last 30 years^44,83^. This observation suggests that the gullies could be related to erosion by density flows induced by the drawdown associated with the depressions (Bernoulli wakes) produced by ships with drafts that reach just few meters above the floor of this canal^84^. This drawdown induces long waves that shoal toward the marsh areas entraining sediment that is then transported by gravity towards the shipping canal likely forming the observed gullies.

### Marine macro-litter

**Figure 6:**
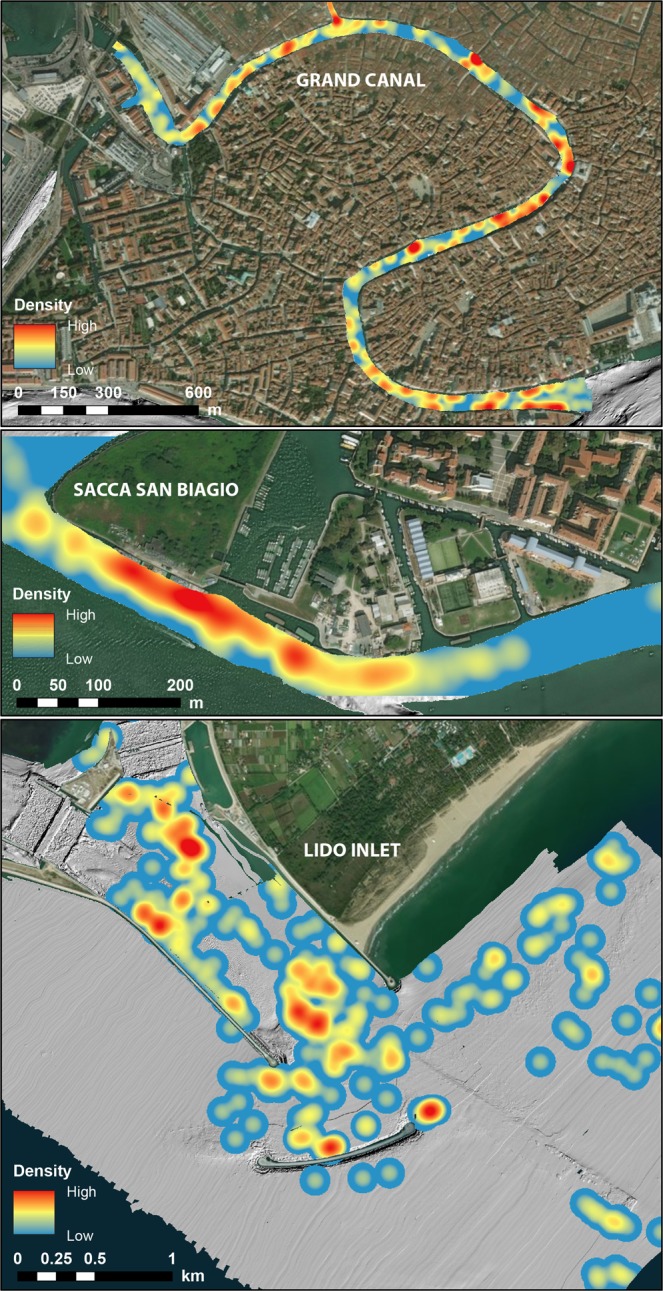
Density of marine macro-litter on the Grand Canal sea-floor, close to the city of Venice and in the Lido Inlet. The map of the whole lagoon is available in the Supplementary material (Fig. A1). Satellite image source: Esri DigitalGlobe, GeoEye, i-cubed, USDA,USGS,AEX,Getmapping, Aerogrid, IGN, IGP, swisstopo, and the GIS User Community, https://services.arcgisonline.com/ArcGIS/rest/services/World_Imagery/MapServer.

Marine macro-litter is present throughout the floor of the channels though with highly variable concentrations mainly depending on the economic activities in the adjacent area. Thanks to the high resolution achieved by the newest generation of MBES mapping, we were able to assess the spatial distribution of the marine macro-litter in the full area of the Venice Lagoon tidal channels and inlets and to estimate their density in terms of items/km^2^. We found that the average density for the entire survey area is equal to 7.5 items/km^2^ and that the highest litter concentration occurs, not surprisingly, around the cities of Venice and Chioggia and at the lagoon inlets. In particular, in the Grand Canal, we found the highest value of mean abundance of marine litter on the sea-floor of 1161 items/km^2^ (Fig. 6). The Grand Canal has never been dredged so far and it probably contains layers of waste dating back to the foundation of Venice. Considering that the dispersion of plastic in the environment worldwide dates back from 1950^85^, a gross estimate of the average rate of the accumulation of rubbish on the sea floor goes from zero (in pristine areas) to ca 20 items per km^2^ yr^−1^ (1161 items per km^2^ in 60 yr in the case of S. Biagio area) (Figs 4f and 5); limitations to this rough estimate come from three facts: a) not all the garbage encountered on the sea floor is plastic and therefore some of it can have been thrown earlier than 1950; b) human impacts are documented to occur at an exponential rate^7^ and therefore a 60 yr average may not offer a realistic view of the ongoing trend; c) only a repetition of the survey in the near future will allow quantification of a likely increase of the use of the sea-floor as a disposal area and definition of its rate.

The presence of numerous objects of anthropogenic origin on the sea-floor alters the sea-floor morphology as well as benthic habitats: it favours the formation of erosional and accretionary bedform patterns that entrain sediment in front of and create a scour behind (down-current of) the object in the direction of the current (obstacle scour), resembling the typical comet marks initially observed in estuaries^86^. At places, and depending on the tidal currents, scouring or comet marks develop at the base of the “bricole” placed on the side of the channels (Fig. 5).

At the same time, the marine macro-litter on the sea-floor may alter the surrounding habitats by providing a new hard substrate (this is the case for example of the rip-rap elements accidentally lost in the tidal inlets, the wrecks and the ancient structures classified as archaeological heritage), potentially covering large portions of the settled communities^87^, causing chemical and physical pollution^88^, and interfering with life on the seabed^89^.

The abundance and distribution of marine litter on the sea-floor is, at the moment, much less widely assessed than at the sea surface. The presence of marine litter on the sea floor is generally investigated by scuba divers in shallow coastal and/or coral reef environments^90,91^, submersible dives^92,93^, remotely operated vehicles (ROVs) in deep waters^94–97^ and by trawl sampling by fishing or research vessels^98–102^. Carefully processed high-resolution MBES data represent an economic and comprehensive tool to extend such studies systematically to broader coastal areas.

In summary, extensive hard structures, small features like poles or pipelines and rip-rap debris, or diffused littering on the sea-floor are all likely to have long lasting effects on the sea floor ecosystem; more important, most anthropogenic impacts can be expected to have an amplifying effect on the ecosystem through time: new substrata are provided to benthic species that otherwise would not settle in the lagoon; and most features condition the flow of tidal currents thereby favouring focused scouring and long lasting erosion and deepening of relevant portions of the lagoon, also with a likely impact on benthic communities.

## Conclusions

While the impacts of human activities on coastal environments have received extensive attention in scientific literature so far, this study for the first time proposes a quantitative and direct assessment of the human footprint on the sea-floor of a shallow coastal lagoon. The new bathymetric data presented in this study highlight the pervasive occurrence of direct and induced anthropogenic imprints and provide: a) unprecedented information locating the hotspots where sea-floor erosion is rapidly advancing in response to a variety of human pressures ranging from coastal engineering (construction of hard structures) to ship traffic; and b) a direct assessment of the mean abundance of marine macro-litter in a large area of the Venice Lagoon and the characterization of marine litter hotspots. The hope is to rise the awareness of stakeholders, decision makers and general public on the hidden anthropogenic imprint that should be taken seriously into account even if less visible than its equivalent on land.

This study provides a benchmark reference to quantitatively evaluate possible short and long-term hydro-morphological changes in the lagoon induced by the presence of large sea-floor anthropogenic structures and by the construction and functioning of the MOSE defence system.

The detailed mapping presented here proposes a new approach that can be adopted in the study of other lagoons and coastal environments worldwide. Moreover, this approach can be adopted to perform impact assessments of human activities on the sea-floor in shallow-marine environments, integrating those currently based only on sparse sampling and indirect estimates of human pressures. An improved knowledge of the human footprint on the morphology of the sub-aqueous part of coastal lagoons lays the foundations for their cost-effective monitoring and sustainable management by offering a means to evaluate the environmental status of the underwater (hidden) structure of the systems.

## Methods

### Data acquisition and processing

The MBES data were collected from May to December 2013 with a Kongsberg EM-2040 DC dual-head system with 800 beams (400 per swath). The MBES was pole-mounted on the bow of the vessel RV Litus, a 10-m long boat with 1.5-m draft. The frequency of MBES was set to 360 kHz. For positioning, a Seapath 300 system was used with the correction of a Fugro HP differential Global Positioning System (dGPS, accurate to 0.20 m), while a motion unit corrected pitch, roll, heave and yaw movements (0.02 degrees roll and pitch accuracy, 0.075 degrees heading accuracy). A Valeport mini SVS sensor was mounted close to the transducers to measure continuously the sound velocity for the beam forming. Sound velocity profiles were systematically collected with an AML oceanographic Smart-X sound velocity profiler. With the Kongsberg native data acquisition and control software SIS (Seafloor Information System) we logged, displayed and checked the data in real-time. CARIS HIPS and SIPS (v.9) was used to account for sound velocity variations, tides and basic quality controls in the derivation of bathymetric data. Backscatter mosaics were created combining the georeferenced backscatter rasters generated by the Geocoder algorithm. Geocoder corrects the system settings, transmission loss, insonification area and incidence angle^103^. In the generation of backscatter rasters the CARIS adaptive Angle Varying Gain (AVG) correction was applied to the raw backscatter data to remove the angular artefacts of sediment from the imagery. The Despeckle option removed isolated pixels^104^. The bathymetric grids were exported from CARIS as text files with grid resolutions ranging from 0.05 to 0.5 m. They were converted to 32-bit raster files using Global Mapper (v12). The raster files were then imported in ArcGIS (v10.2)^105^ for further analysis^37^. Ground-truth samples, which included scuba-diving operated HD video transects, drop-frame photo-quadrants and biological and sediment samples, were collected in 2014–2015. The areas selected for ground-truth tests were surveyed again with MBES in order to assure that no substantial change had occurred since the survey in 2013. The water circulation in the lagoon, induced by tide, wind, and seasonal water changes, heat and salt fluxes, was simulated by the unstructured model SHYFEM^106^. Using unstructured numerical meshes composed of triangular elements of variable form and size, the model reproduces adequately the complicated geometry and bathymetry of the Venice Lagoon. The model runs in a 3-D baroclinic mode, using observed forcing and boundary conditions (i.e. wind stress, heat and salt fluxes, precipitation, sea level and freshwater discharge). Comparing model results with sea level data measured from all tide-gauge stations present in the lagoon, we estimated an error of about 2 cm for the simulated water level. Water levels simulated by the numerical model SHYFEM were also used to correct MBES bathymetric data for tidal oscillations. All the corrections are referred to the Venice local datum Punta Salute 1897 that lays about 26 cm below the mean sea level.

### DEM analysis

The bathymetric data were processed and combined to generated a detailed digital bathymetric map (DEM) of the channel sea-floor (Fig. 1). The DEM was analyzed to map all detectable morphological features related (directly or indirectly) to anthropogenic activities on the sea-floor using the ArcGIS 10.2 software, according to the categories shown in Fig. 4. The features were visually identified and digitized as polygons when their dimensions were larger than a minimal spatial unit set to 10 m. Below this minimal spatial unit, the features were saved in a point feature shape file, recording their dimensions. Around each point a buffer of 1.5 m (average feature dimension recorded in the geodatabase) was created to transform the points in polygons. To help the digitalization process and to make it more objective, the main terrain attributes^107,108^ were extracted from the bathymetry: slope, broad Benthic Position Index (BPI) and Ruggedness. The BPI and ruggedness were calculated with BTM^109^. BPI and slope were useful to identify the hard structure scour holes (see Fig. A2 Supplementary Material in the supplementary material and Ferrarin *et al*.^110^ for the full workflow for scour identification) and dredging areas, respectively, whereas high ruggedness values helped to highlight the rip-rap areas and the presence of small objects (see Fig. A2). The classification of the features was checked at different stages by different people in the group in order to make it less subjective and more consistent. The computation of the mean marine litter abundance on the sea-floor was done by calculating the number of point anthropogenic morphological features (items) identified as macro-litter in the survey area. Two independent operators validated the identification to minimize subjective interpretations. The presence of marine litter was evaluated both in terms of occurrence (frequency of marine litter types) and density (marine litter items/100 m^2^). The Kernel Density tool of ArcGIS allowed the characterization of the regions of accumulation of litter, calculating the density of point features around each output raster cell (Fig. 6a). The kernel function is based on the quartic kernel function^111^. For the Grand Canal and the new channel of Fusina (close to Sacca San Biagio) search radius of 30 m considering cells of 0.5 × 0.5 m (Fig. 6b). For the entire lagoon (including the inlets) we applied a search radius of 100 m and a cells of 5 × 5 m.

## Supplementary information

Supplementary Material
