# Supplementary material for: Assessing the human footprint on the sea-floor of coastal systems: the case of the Venice Lagoon, Italy
Source: Sci Rep. 2019 Apr 29;9:6615. doi: 10.1038/s41598-019-43027-7 (PMC6488697; doi:10.1038/s41598-019-43027-7)
Supplement: Supplementary file 1 — Supplementary Material [file 41598_2019_43027_MOESM1_ESM.pdf]

# Assessing the human footprint on the sea-floor of coastal systems: the case of the Venice Lagoon, Italy

**Fantina Madricardo[1,*], Federica Foglini [2], Elisabetta Campiani[2], Valentina Grande[2], Elena Catenacci[2], Antonio Petrizzo[1], Aleksandra Kruss[1], Carlotta Toso[1], and Fabio Trincardi[1,*]**

[1]CNR-National Research Council, ISMAR-Marine Sciences Institute in Venice, Castello 2737/f, 30122 Venice, Italy
[2]CNR-National Research Council, ISMAR-Marine Sciences Institute in Bologna Via Gobetti, 101, 40129 Bologna, Italy
[*]corresponding authors: fantina.madricardo@ismar.cnr.it; fabio trincardi@cnr.it

## ABSTRACT

Coastal systems are among the most studied, most vulnerable, and economically most important ecosystems on Earth; nevertheless, little attention has been paid, so far, to the consequences of human activities on the shallow sea-floor of these environments. Here, we present a quantitative assessment of the effects of human actions on the floor of the tidal channels from the Venice Lagoon using 2500 kilometres of full coverage multibeam bathymetric mapping. Such extended dataset provides unprecedented evidence of pervasive human impacts, which extend far beyond the well known shrinking of salt marshes and artificial modifications of inlet geometries. Direct and indirect human imprints include dredging marks and fast-growing scours around anthropogenic structures built to protect the historical city of Venice from flooding. In addition, we document multiple effects of ship traffic (propeller-wash erosion, keel ploughing) and diffuse littering on the sea-floor. Particularly relevant, in view of the ongoing interventions on the lagoon morphology, is the evidence of the rapid morphological changes affecting the sea-floor and threatening the stability of anthropogenic structures.

## A Supplementary material

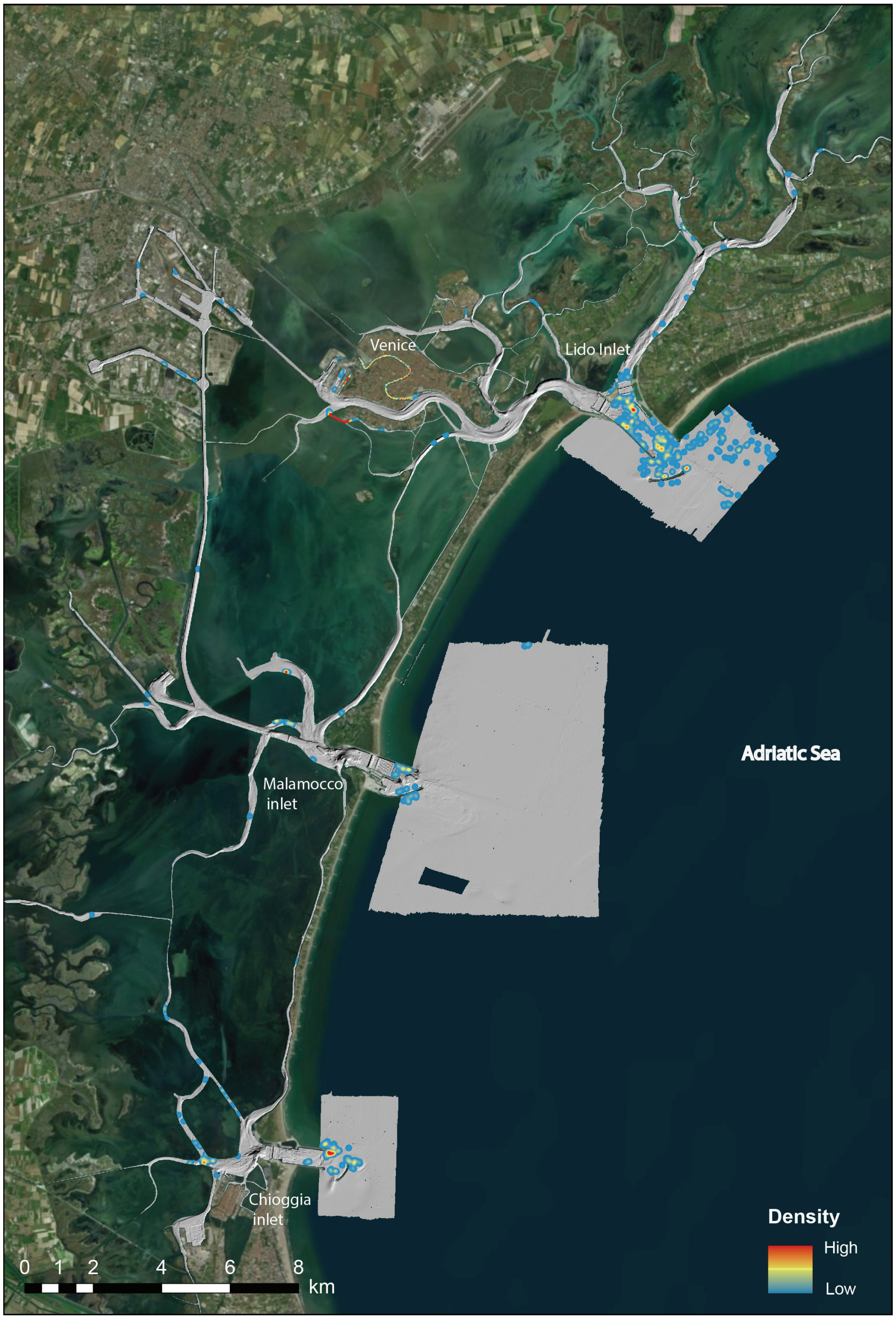


**Figure A.1.** Map the density of the marine macro-litter on the sea-floor of the Venice Lagoon tidal channels and inlets. Satellite image source: Esri DigitalGlobe, GeoEye, i-cubed, USDA,USGS,AEX,Getmapping, Aerogrid, IGN, IGP, swisstopo, and the GIS User Community,
https://services.arcgisonline.com/ArcGIS/rest/services/World_Imagery/MapServer

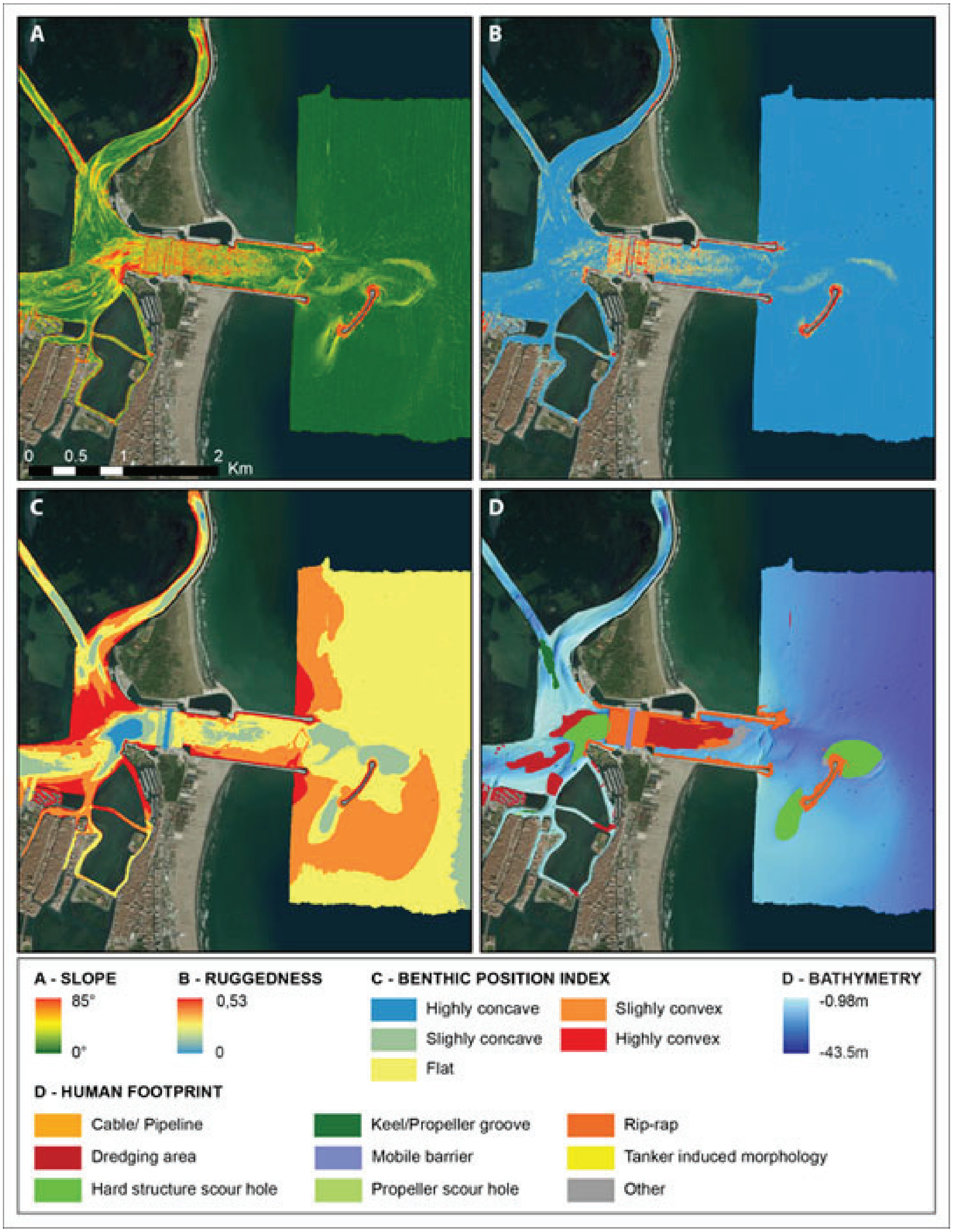


**Figure A.2.** Map of: a) the bathymetry and the human footprint in the Chioggia inlet (localized as in Figure 4a); b) the slope, c) the BPI (calculated with inner and outer radius of 50 and 750, respectively) and d) the ruggedness (calculated with radius 5) extracted from the bathymetry to support the visual interpretation and digitalization of the sea-floor anthropogenic morphologies. Satellite image source: Esri DigitalGlobe, GeoEye, i-cubed, USDA,USGS,AEX,Getmapping, Aerogrid, IGN, IGP, swisstopo, and the GIS User Community, https://services.arcgisonline.com/ArcGIS/rest/services/World_Imagery/MapServer

- The MBES data are available from the repository: Madricardo, F., Foglini F and. Trincardi F. Marine Geosciences Data System. http://dx.doi.org/10.1594/IEDA/323605
- The historical maps of the Venice Lagoon belonging to the CNR-ISMAR Cartographic Heritage were digitized and are available online: http://cigno.ve.ismar.cnr.it/maps/1200
- The Multiple and pervasive human impacts in coastal lagoons literature review is a Research Object that can be found at the link: http://sandbox.rohub.org/rodl/ROs/multiple_and_pervasive_human_impacts_in_coastal_lagoons_literature_review-release-1/
